# High-speed running and sprinting in professional adult soccer: Current thresholds definition, match demands and training strategies. A systematic review

**DOI:** 10.3389/fspor.2023.1116293

**Published:** 2023-02-13

**Authors:** Antonio Gualtieri, Ermanno Rampinini, Antonio Dello Iacono, Marco Beato

**Affiliations:** ^1^ Sport Science and R&D Department, Juventus Football Club, Torino, Italy; ^2^ School of Health and Sports Sciences, University of Suffolk, Ipswich, United Kingdom; ^3^ Human Performance Laboratory, MAPEI Sport Research Centre, Olgiate Olona, Italy; ^4^ Sport and Exercise Discipline Group, Human Performance Research Centre, Faculty of Health, University of Technology Sydney, Moore Park, NSW, Australia; ^5^ Division of Sport and Exercise, School of Health and Life Sciences, University of the West of Scotland, Hamilton, United Kingdom

**Keywords:** football, GNSS, GPS, velocity thresholds, team sports, elite sports

## Abstract

The aims of this systematic review were (1) to summarize the evidence on absolute velocity thresholds used to classify high-speed running and sprinting, (2) to examine the existing evidence about the individualized thresholds approach, (3) to describe high-speed and sprint running distance match demands, and (4) to provide training strategies for eliciting HSR and sprinting during training sessions in professional adult soccer. This systematic review was conducted following the PRISMA 2020 guidelines. After the authors' screening, 30 studies were included in this review. This review found that, to date, there is no consensus on the absolute thresholds defining high-speed and sprint running in adult soccer players. Until international standards are defined, it is reasonable to set absolute thresholds considering the range of values found in the literature collected in this review. Relative velocity thresholds could be considered for specific training sessions whose goal is to reach near maximal velocity exposure. During official matches, high-speed and sprint running distances ranged from 911 to 1,063 m and 223–307 m, respectively, in professional female soccer players, while ranges from 618 to 1,001 m and 153–295 m, respectively, in professional male soccer players. During training, game-based drills designed in formats using relative areas per player greater than 225 m^2^ and 300 m^2^ appear to be adequate for achieving high-speed running and sprinting exposure, respectively, for male players. The combination of game-based, running exercises and soccer circuit-based drills is advisable to ensure adequate high-speed and sprint running exposure both at a team and individual level.

## Introduction

Soccer is a physically demanding team-sport characterized by an intermittent activity profile with high-intensity activities such as accelerations, decelerations, changes of direction, sprinting, jumping, and tackling interspersed by low-intensity phases of passive (i.e., standing) and active recovery (e.g., walking, jogging) (1, 2). The match play intensity in male soccer has considerably increased over the last 15 years, especially due to the greater high-speed running (HSR) (distance covered at speeds between 19.8 km·h^−1^ and 25.1 km·h^−1^ increased ∼29%) and sprint (distance >25.1 km·h^−1^ increased ∼50%) locomotive demands, which now account for ∼7%–11% and ∼1%–3% relatively to the total distance covered during a match, respectively (2–4). Similarly, intense running in female soccer has increased across various playing positions by approximately 16%–32% from the 2015 to the 2019 *Fédération Internationale de Football Association* (FIFA*)* World Cup (5). The evolution of soccer matches intensity implies that players should be adequately prepared to cope with the physical demands of the game. Furthermore, HSR and sprint activities are also considered as key determinants for successful performance (6). To illustrate, straight sprinting has been identified as the single most frequent locomotive action preceding goal situations, performed by either the scoring player or the assisting one (7, 8). Moreover, there is evidence highlighting significant positive associations between HSR and sprint distances covered by players in specific positions (e.g., wide midfielders and forwards) and the number of matches won by their team (9). Accordingly, the ability to sustain HSR and sprinting can be considered a key characteristic for soccer players to compete at the professional level (10). Therefore, developing players' capacity to perform HSR and sprinting is paramount for the coaching staff and sport science departments in professional soccer.

In the past, low velocity thresholds (i.e., 14.4 km·h^−1^–15 km·h^−1^) were selected to define HSR and sprinting. That was due to the low reliability of wearable micro-technologies such as Global Navigation Satellite Systems (GNSS) and video tracking systems devices available at those times, usually sampling at frequencies lower than 5 Hz (11–13). The advances in these tracking systems have enabled a more accurate quantification of soccer matches and training loads for activities performed at higher velocity (14, 15). At present, the available GNSS technology is deemed valid for measuring distances covered at HSR and peak velocity in sports (16) as well as reliable with excellent inter-unit reliability reported for linear sprint distances [coefficient of variation (CV) = from 1.64% to 2.91%] (17) and sport specific circuits (14). Consequently, tracking technologies are now more commonly used for monitoring HSR and sprinting distances during training and competitions in soccer (18). Despite this widespread use, the current practices among soccer practitioners and sport scientists are not exempt of limitations especially due to the non-standard definitions of HSR and sprinting and the relative velocity thresholds set for their quantification (19). Nowadays, while the official reference thresholds in official competitions of soccer governing organizations such as the *Union of European Football Associations* (UEFA) and the FIFA are 19 km·h^−1^ and 23 km·h^−1^ and 20 km·h^−1^ and 25 km·h^−1^ for HSR and sprinting in women and men, respectively, a large heterogeneity emerges from the scientific literature (5, 20). Therefore, a systematic review that summarizes the evidence on velocity thresholds reference values specifically for professional female and male soccer is needed. The unfolding evidence would facilitate data and knowledge sharing between sport science departments and possibly foster the design of multicentric studies involving clubs from different countries, allowing less uncertain and more robust conclusions to be drawn.

More recently, the use of individual relative thresholds has been proposed as an alternative approach to arbitrary velocity thresholds selection for better quantifying external load measures in soccer (18). For example, in a recent study comparing external loads between starting and non-starting players during a 21-day congested fixture period of a Serie A team, significant between-group differences for sprint distance emerged only when individualized thresholds (i.e., 80% of the maximum peak velocity) were used. This may suggest that the selection of velocity thresholds should account for the individual maximal velocity to accurately quantify sprint distance outcomes during training and matches (21). Nevertheless, given that only preliminary evidence is available on this topic, further research is warranted to investigate the effectiveness of using individual relative thresholds in soccer.

The monitoring of HSR and sprinting distance has been traditionally used to inform training practices with the aim to physically prepare soccer players to the match demands. However, some training contents and drills are unable to elicit HSR or sprinting: summarizing the literature pertaining HSR and sprinting demands and outcomes across different types of exercises can allow practitioners to make evidence-informed decisions when planning training sessions aimed at ensuring adequate HSR and sprint distances exposure.

Therefore, the aims of this systematic review were: (1) to summarize the evidence on velocity thresholds used to classify HSR and sprinting in adult professional female and male soccer players, (2) to examine the existing evidence about the use of individualized thresholds, (3) to describe the HSR and sprinting demands during soccer matches, and (4) to provide training strategies for eliciting HSR and sprinting during training sessions.

## Methods

The Preferred Reporting Items for Systematic Reviews and Meta-Analyses Protocols (PRISMA) statement was consulted prior to the start of this review and the checklist completed (22). The review methods were established prior to the conduct of the review (including review question, search strategy and inclusion/exclusion criteria) and no significant deviations from the protocol were made. For this review, an assessment of the risk of bias was not performed since the complexity of judging the quality of observational studies (23).

### Search methods for identification of studies

The same systematic search was performed in PubMed (MEDLINE), Web of Science and SPORTDiscus (EBSCO) until October 2022 with no restriction for year of publication. The following search strategy adapted for each database was used: ((“football” OR “soccer”) AND (“adult” OR “senior”)) AND ((“high speed” OR “sprint”) AND (“running” OR “distance” OR “effort”)) AND (((“match” OR “game”) AND (“demand” OR “request”)) OR (“training” OR “session”)) (Table 1).

**Table 1:** Search strategy.

Variable	Search terms
Population	(“football” OR “soccer”) AND (“adult” OR “senior”)
Load	(“high speed” OR “sprint”) AND (“running” OR “distance” OR “effort” OR “velocity”)
Variable	((“match” OR “game”) AND (“demand” OR “request”)) OR (“training” OR “session”)
Final search	Combination of the three groups: “Population” AND “Load” AND “Variable”

In addition, manual searching, and reference checking have been performed by three independent reviewers (AG, MB and ER) to search other relevant reports.

### Inclusion and exclusion criteria

Studies were included if they met the following criteria: (1) original research article; (2) the study was published in English and in a peer-reviewed journal; (3) the research design was either an observational study or an intervention study including a control group; (4) participants were professional soccer players of any sex and ≥18 years of age; and (5) the study reported HSR or sprint distances outcomes, defined according to arbitrary or individualized velocity thresholds and collected during official matches or training sessions. Manuscripts were excluded from the review in any of the following cases: (1) sport or football code was different from 11v11 soccer (e.g., American and Australian Football); (2) the subjects played at a lower level of the third national league (if not defined as professional players); (3) metrics reported did not include HSR and sprinting values; (4) GNSS sample frequency used in the study was under 5 Hz, since HSR and sprinting distances have been shown to be less accurate and reliable when tracked with 5 Hz units (12, 13); (5) data came from manual coding.

### Data collection and analysis

Two reviewers (AG and MB) independently assessed titles and abstracts of all identified articles, which were downloaded into a web app for systematic reviews (rayyan.qcri.org, Hamad Bin Khalifa University, Qatar) (24). A third independent reviewer was consulted to settle conflict (ER).

### Data extraction

Two reviewers (AG and MB) independently extracted data from all relevant articles by reading the articles in full. Key areas of interest were elucidated, and the information extracted included:

• Study population (sample size, gender, competition level and Club's name when available).
• Number of training sessions or weeks, number of games, number of seasons included in the study.
• High-speed and sprint running metrics, adopted absolute and/or individualized thresholds.
• Details from the study (main findings, average training or match values about physical demand).

## Results

### Search results

The systematic search through the 3 databases (i.e., Pubmed, Web of Science, SPORTDiscus) produced 823 records, which were screened using a web app for systematic reviews (rayyan.qcri.org, Hamad Bin Khalifa University, Qatar) (24) to remove any duplications. The summary of the systematic search was as follows:

- 697 results on Pubmed
- 76 results on Web of Science
- 50 results on SPORTDiscus

**Figure 1:**
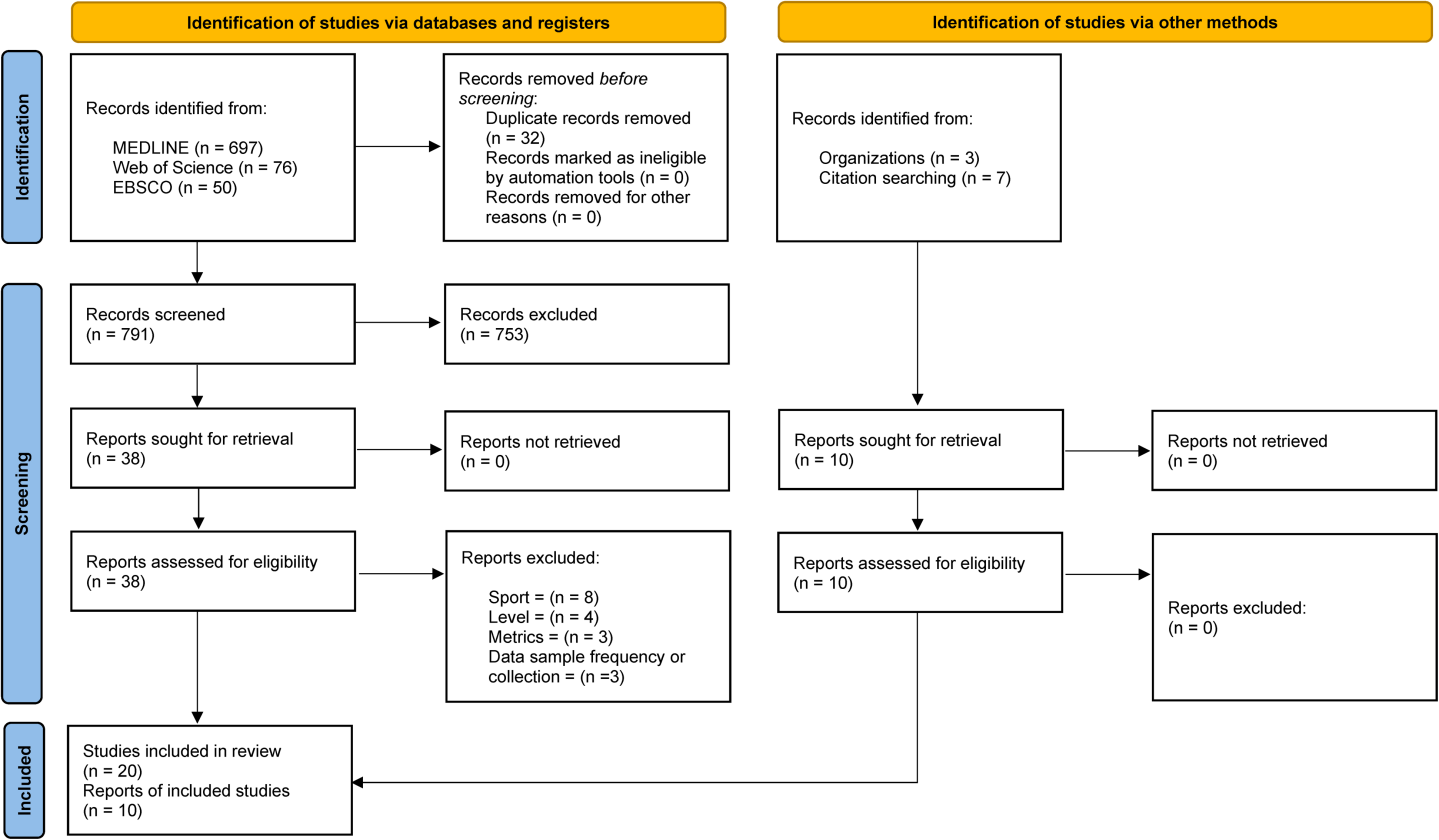
PRISMA flow diagram for the description of the overall process.

After removing duplicates (*n* = 32), to enable simultaneous screening against the inclusion–exclusion criteria, titles and abstracts were screened to remove articles that were clearly not relevant. At this stage, 753 records were excluded. The full texts of the remaining 38 articles were then accessed for complete screening with 18 studies being excluded as did not meet the inclusion criteria. Ten additional studies were found through other sources, 3 from authors' archives and 7 following references screening of the 38 articles accessed. Independent screening results were then combined, and any disagreements was resolved by consensus discussion between the authors (AG, MB, and ER). After the final screening, 30 studies were included in this systematic review. The PRISMA flow diagram for the description of the overall process is reported in Figure 1.

### Descriptive characteristics of the included studies

After final screening, 1 longitudinal observational study and 29 observational studies were included in the systematic review. Data regarding sample size, gender, age, load metrics and results about match and training demand were extracted, verified for accuracy, and reported in Table 2.

**Table 2:** Summary of studies accompanied by study design, subjects, high-speed running metrics reported and details from the studies.

References	Participants	HSR metrics	Details
Scott et al., 2013 (25) *Observational study*	Professional male soccer players (*n* = 15) Individual training sessions (*n* = 97)	HSR > 14.4 km·h^−1^ VHSR > 19.8 km·h^−1^	Absolute and % of total distance values recorded during training: HSR = 544 ± 255 m (12.0 ± 3.8%), range 106–1,343 m (4.9–23.3%) VHSR = 132 ± 101 m (2.8 ± 1.9%), range 7–541 m (0.2–8.8%)
Wehbe et al., 2014 (26) *Observational study*	Elite male adult soccer players from Australian-league (A-League) soccer (Sydney Football Club) (*n* = 19) Preseason matches (*n* = 8)	HSR > 19.7 to ≤25.1 km·h^−1^ Sprint > 25.1 km·h^−1^ Putting together thresholds: HIR > 14.3 km·h^−1^ VHIR > 19.7 km·h^−1^	Positional comparison: midfielders covered 28% more HIR distance than defenders. Match half comparison: HIR and VHIR decreased from the first to the second half by 10 and 11%, respectively. Match status analysis: when the team was winning, average speed was 4% lower than when the team was drawing (*p* ≤ 0.05, *d* = 0.32). Pre- and post-goal analysis: scoring or conceding goals did not appear to affect HIR. In the 5-minute intervals before and after a goal was scored, 5-minute HIR distance was 140 and 128 m, respectively (*p* = 0.464). In the 5-minute intervals before and after a goal was conceded, 5-minute HIR distance was 144 and 110 m, respectively (*p* = 0.015). Average and peak 5-minute HIR distance during the whole match was 123 and 237 m, respectively.
Malone et al., 2015 (27) *Observational study*	Professional male players from English Premier League (Liverpool) (*n* = 30) Preseason weeks (*n* = 6) In-season weeks (*n* = 36) Microcycles (*n* = 3)	HSD > 19.8 km·h^−1^	Higher total distances covered in the early stages of the competitive season and the highest HR response occurring at the midpoint of the season. HSD 1-week in-season microcycles (daily means): early-season = 243 ± 229 m, mid-season = 225 ± 213 m, late-season = 146 ± 104 m. Wide midfielders covered a higher amount of HSD across the different microcycles than central defenders (94 [43–145] m, ES = 0.47 [0.22–0.73], small). Periodization of training load was typically confined to MD-1 (regardless of mesocycle), whereas no differences were apparent during MD-2 to MD-5.
Anderson et al., 2016 (28) *Observational study*	English Premier League male players (*n* = 12) Training sessions (*n* = 10) + matches (*n* = 6) (1-, 2-, 3-game weeks)	HSR = 19.8–25.1 km·h^−1^ Sprint > 25.1 km·h^−1^	The majority of distance during specific training sessions was completed in the low-to moderate speed zones, whereas the distance completed in high-intensity zones were largely completed in the game itself. HSR: match demand = 706 m; training stimulus = 156 m (1-game week), 192 m (2-game week), 81 m (3-game week). Sprinting: match demand = 295 m; training stimulus = 8 m (1-game week), 16 m (2-game week), 7 m (3-game week).
Carling et al., 2016 (29) *Observational study*	French League 1 male players (*n* = 12) Matches (*n* = 31)	HSR = 19.8–25.2 km·h^−1^ Sprint > 25.2 km·h^−1^ Total HSR (THSR, ≥19.8 km·h^−1^);	Math demand: HSR = 587 ± 133 m; Sprint = 184 ± 87 m; THSR = 770 ± 206 m.
Chmura et al., 2017 (10) *Observational study*	International male soccer players from 32 teams (*n* = 340) Single observations during 2014 World Cup (*n* = 905)	HIR = 19.9–25.2 km·h^−1^ (% of TD) N° of sprints >25.2 km·h^−1^	The mean distance covered by players at high intensity was 8.83 ± 2.11%. It was significantly longer between the quarter-finals and the semi-finals (*p* ≤ 0.01). In the semi-finals the percentage values of TD covered at HI were the greatest. Individually, the greatest percentage achieved was 17% by 2 midfielders. The mean number of sprints performed was 33 ± 11, 1 every 173 s. The greatest number of performed sprints was 68, 1 every 82 s, in a semi-final match. Winning a soccer championship requires players to run longer mean total distances and longer distances at high intensity during a single match.
Mara et al., 2017 (30) *Observational study*	Elite female players from the Australian national league (W-League) (*n* = 12) Matches (*n* = 7)	HSR = 12.24–19.0 km·h^−1^ Sprint > 19 km·h^−1^ High Speed Runs and Sprints (n)	Match demand: HSR = 2,452 ± 636 m; Sprint = 615 ± 258 m; high-speed runs = 376; sprints = 70. A large proportion of high-speed runs (81–84%) and sprints (71–78%) were performed over distances less than 10 m, with 14 s between high-speed runs and 87 s between sprints. The characteristics of high-speed runs and sprints differed between repeat and nonrepeat efforts, and the activity profiles of players varied according to positional groups and period of the match.
Miñano-Espin et al., 2017 (31) *Observational study*	Real Madrid matches (*n* = 149): data from Real Madrid and opposing teams’ male players	HIR = 21.1–24.0 km·h^−1^ Sprint > 24 km·h^−1^ High Speed Runs and Sprints (n)	Match demand: HIR distance = 269 m Real Madrid vs. 285 m opposing team; Sprint distance = 245 m vs. 248 m; High Intensity Runs = 11; Sprints = 20. Players from Real Madrid covered shorter distances in HIR and Sprint and executed less sprints than players from the opposing team. No differences were revealed in the HIR and Sprint distances or the number on high intensity runs and sprints performed by players from Real Madrid depending on the quality of the opposition.
Abbott et al., 2018 (32) *Observational study*	Premier League 2 under 23 professional male players (Brighton and Hove Albion) (*n* = 46) Matches (*n* = 22) LSG, MSG, SSG (*n* = 39)	VHSR = 100% MAS – 30% ASR Sprint >30% ASR Mean and 1-min peak values	Despite eliciting significantly higher average total distances compared with competition, LSGs produced significantly lower peak total distance relative to the competition. For VHSR and sprinting, LSGs elicited similar average intensities to competition; however, peak intensities were significantly lower than competition. VHSR and sprinting distances increased with game format, with LSGs (>7v7) producing the highest intensities. Only LSGs were able to replicate competitive demands, with SSGs and MSGs significantly below competitive values for all positions.
Baptista et al., 2018 (33) *Observational study*	Professional male soccer players (Tromsø Idrettslag) (*n* = 18) Official matches (*n* = 23)	HIR ≥ 19.8 km·h^−1^ Sprint ≥ 25.2 km·h^−1^ Number of HIR and sprint efforts of various length (1–5, 6–10, 11–15, 16–20, 21–25, 26–30, 31–35, 36–40, 41–45, 46–50 m) CoD counts	CB had the lowest values of all positions in both variables but especially pronounced in Sprint (1 m·min^−1^) when compared with CF (2.5 m·min^−1^). HIR analysis: CF presented higher values in 26-30 m than all the other positions, while distances of 36–40 and 46–50 m were covered more times by FB. CB were the players with lowest values in these longer distances (36–40 and 46–50). Sprint analysis: CB, FB, CM and WM performed higher number of 1–5 m sprints, while CF covered higher number of 6–10 m sprints. The most common distance covered in HIR for CB, CM, WM and CF was 1–5 m, but for FB was 6–10 m.
Malone et al., 2018 (34) *Longitudinal observational study*	Professional male soccer players (Benfica) (*n* = 37) Weeks (*n* = 48)	HSR > 14.4 km·h^−1^ Sprint > 19.8 km·h^−1^	When HSR and SR distances are considered independently of aerobic fitness and previous training load history, a U-shaped association exists for distance completed at these speeds and subsequent injury risk. Players with higher aerobic fitness were able to complete increased weekly HSR and SR distances with a reduced injury risk. Higher 21-day chronic sRPE-TL (≥2,584 AU) allow exposure to greater volumes of HSR and SR, which in turn offers a protective effect against injury. 1-week safer zone: HSR = 700–750 m, SR = 200–350 m. Absolute weekly change safer zone: HSR < 100 m, SR < 50 m 3:21 ACWR safer zone: HSR < 0.85, SR = 0.71–0.85
Scott and Lovell, 2018 (35) *Observational study*	International women's soccer players (*n* = 22)	HSR > 12.67 km·h^−1^ (HRDP) VHSR > 17.82 km·h^−1^ (MAS)	In this approach, each players running speed corresponding to HRDP, together with their MAS determined from the VAM-EVAL, were used as the entry-points to the HSR and VHSR zones. Individualised speed thresholds for external load monitoring were not able to better quantify the dose-response of football training during a 21-day training camp in players representing the highest level of women's football. Quantifying the external load using players’ peak sprinting speed demonstrated a lower capacity to determine the dose-response of training, with consistently lower associations with heart rate and RPE.
Martín-García et al., 2018 (36) *Observational study*	Professional male soccer players (Barcelona 2nd team) (*n* = 24) Matches (*n* = 37) + training weeks (1 game per week) (*n* = 42)	HSR > 19.8 km·h^−1^ Sprint > 25.2 km·h^−1^	When comparing starters and non-starters at MD + 1, thanks to the SSG approach used in players with limited game time, non-starters demonstrated greater external loads for TD, HMLD, AMP, ACC, and DEC, but not for HSR or SR. The session that produced the greatest HSR (43%) and SR (45%) distances relative to competition was MD-4. HSR and SR distances are the metrics illustrating the most variability within the microcycle (>80%), which is consistent with the variability found in SSG formats (60–140%), but lower than competition variability (20–30%).
Martín-García et al., 2018 (37) *Observational study*	Professional male soccer players (Barcelona 2nd team) (*n* = 23) Official matches (*n* = 37)	HSR > 19.8 km·h^−1^ Sprint > 25.2 km·h^−1^ 1′, 3′, 5′ and 10′ MIP using TD, HMLD e AMP as the criterion variables	HSR: FB covered the greatest distance, reaching values of 47.2 ± 24.0 m·min^−1^ in the 1′ period. 1′ MIP demand using TD as the criterion variable (positions’ average): TD = 191.6 ± 19.7, HSR = 38.3 ± 23.1, Sprint = 10.6 ± 15.6, ACC > 3 m·s^2^ = 2.8 ± 1.6, DEC < −3 m·s^−2^ = 3.5 ± 1.6 1′ MIP demand using HMLD as the criterion variable (positions’ average): TD = 173.5 ± 26.0, HSR = 49.9 ± 19.8, Sprint = 16.6 ± 17.4, ACC > 3 m·s^2^ = 3.5 ± 1.7, DEC < −3 m·s^−2^ = 3.6 ± 1.7
Soroka, 2018 (38) *Observational study*	2010 World Cup male players (*n* = 599)	HIR = 19.9–25.2 km·h^−1^ Sprint > 25.2 km·h^−1^	The largest amount of HIR and Sprint distance was found in midfielders, which did not correspond to studies carried out on players of the Premier League and Primera Division in 2006–2007 (strikers covered the largest sprint distance) (Carling 2008).
Clemente et al., 2019 (39) *Observational study*	Professional male soccer players (Portuguese Second League) (*n* = 23) 5v5 + GK in 40 × 31 m (124 m^2^) 6v6 + GK in 45 × 32 m (120 m^2^) 9v9 + GK in 70 × 50 m (194 m^2^)	Running = 14–20 km·h^−1^ Sprinting > 20 km·h^−1^	Greater values for sprinting distance were found in the full match compared to 5vs5 + GK (*d* = 3.673, strong effect), 6vs6 + GK (*d* = 2.606, moderate effect) and 9vs9 + GK (*d* = 1.903, moderate effect) sided games. MSG are not appropriate for simulating the sprinting conditions of official full matches. LSG (9vs9 + GK) simulate official full matches more accurately than the other sided-games that were studied (5vs5 + GK and 6vs6 + GK).
Clemente et al., 2019 (40) *Observational study*	Professional male soccer players (Sporting Lisbona) (*n* = 27) Training weeks (with 3-4-5 training sessions + 1 game) (*n* = 22)	RD = 14.0–19.9 km·h^−1^ HSR = 20.0–24.9 km·h^−1^ Sprint > 25.0 km·h^−1^ TMr = Training/Match ratio	It was observed that specific variables (e.g., HSR distance and sprinting distance) were associated with substantially lower ratios than other variables. The TMr for RD and HSR distance were 1.2 ± 0.7 and 1.1 ± 0.8, respectively, in 3-days week and 2.3 ± 1.3 and 2.3 ± 1.5, respectively, in 5-days week. This suggests that the number of training sessions tend to emphasize the stimuli of overall distance and that the demand of three days of training is very similar to the demand of one match. Some determinant external load measures (e.g., HSR or sprinting) are clearly undertrained comparing with more prevalent measures (e.g., TD, ACC or DEC): SSG increase the frequency of ACC/DEC while decreasing opportunities to perform HSR or sprinting.
Dalen et al., 2019 (41) *Observational study*	Male soccer players from an elite Norwegian league team (*n* = 26) Matches (*n* = 18) SSGs (28 4vs4 + 28 6vs6) (*n* = 56)	HIR > 19.8 km·h^−1^ Sprint > 25.2 km·h^−1^	HIR (m·min^−1^) in match peak (5 min most demanding period), match mean, 4v4 and 6v6 = 19 ± 3.5, 8.3 ± 2.1, 2.7 ± 0.9, 3.7 ± 2.1. Sprint = 8.8 ± 4, 1.7 ± 0.7, 0.1 ± 0.1, 0.2 ± 0.5. The smaller pitch used for SSGs may lead to a different work pattern from match play, which is supported by the relatively low HIR and sprint distances observed during SSGs in this study. 4vs4 games are a good method of training acceleration and player load tolerance, but SSGs do not represent a good method of training HIR.
Hills et al., 2019 (42) *Observational study*	Championship male soccer players (Hull City Tigers) (*n* = 17) Matches (35 single observations) (*n* = 13)	MSR > 14.4 ≤ 19.8 km·h^−1^ HSR > 19.8 ≤ 25.2 km·h^−1^ Sprint > 25.2 km·h^−1^	Relative TD (+13.4 m·min^−1^) and HSR (+0.4 m·min^−1^) distances covered during rewarm-ups increased with proximity to pitch-entry. Very few HSR and no sprint distance were performed during each warmup or rewarm-up bout. Substitutes covered greater TD (+67 to +93 m) and HSR (+14 to +33 m) distances during the first 5 min of match-play versus all subsequent epochs.
Jones et al., 2019 (43) *Observational study*	Professional male soccer players (English Football League One) (*n* = 37) Matches partitioned in 3 fixture congestion scenarios (*n* = 79)	HID = 19.9–25.2 km·h^−1^ Sprint > 25.2 km·h^−1^	The Linear Mixed Model did not identify significant interactions between position, fixture congestion scenario and time period (*p* = 0.549), position and fixture congestion scenario (*p* = 0.481), nor fixture congestion scenario and time period (*p* = 0.162).
Modric et al., 2019 (44) *Observational study*	Professional male soccer players from Croatian Soccer League (6th of 10) (*n* = 101) Matches (*n* = 14)	RD = 14.4–19.7 km·h^−1^ HSR = 19.8–25.1 km·h^−1^ Sprint > 25.2 km·h^−1^ InStat technical index	Math demand: HSR = 462 ± 160 m; Sprint = 156 ± 97 m. Association between the running performance of players involved in certain playing positions and overall game performance (InStat index). Specifically, it seems that CD distance in the running zone and number of high-intensity accelerations, FB number of decelerations, and FW sprinting distance are crucial physical requirements of team success.
Oliveira et al., 2019 (45) *Observational study*	Elite male soccer players participating in UEFA Champions League (*n* = 19) Weeks (*n* = 39) + matches (*n* = 50)	HSD > 19 km·h^−1^ Hooper Index	Although there are some significant differences between mesocycles, there was minor variation across the season for the internal and external TL variables used. MD-1 presented a reduction of external TL during in-season match-day-minus training comparison.
Park et al., 2019 (46) *Observational study*	International female players (*n* = 27) International matches (*n* = 52)	HSR: ≥ 12.5 km·h^−1^ VHSR ≥ 19 km·h^−1^ Sprint ≥ 22.5 km·h^−1^	PS in elite women = 29.0 ± 1.5 km·h^−1^ *k*-means clustering and Gaussian mixture modelling were not appropriate for soccer given the limited instances in which players move at velocities associated with sprinting, which are often considered key physical performance indicators. A spectral Clustering technique with application of a *β* = 0.1 smoothing factor derived new thresholds featuring both logical validity and analysis rigor. Similar analyses may be warranted to determine appropriate velocity zones for other sports and youth populations.
Rago et al., 2019 (47) *Observational study*	Italian Serie B male soccer players (*n* = 13)	MSR = arbitrary 14.4–19.8 km·h^−1^ or individualised 80–99% MAS HSR = 19.9–25.1 km·h^−1^ or 100% MAS – 29% ASR Sprint = ≥25.2 km·h^−1^ or ≥30% ASR	Perceptual responses (RPE) were moderately correlated to MSR and HSR quantified using the arbitrary method (*p* < 0.05; *r* = 0.53–0.59). However, the magnitude of correlations tended to increase when the individualised method was used (*p* < 0.05; *r* = 0.58–0.67). Distance covered by sprinting was moderately correlated to perceptual responses only when the individualised method was used (*p* < 0.05; 0.55 [0.05; 0.83] and 0.53 [0.02; 0.82]). The magnitude of the relationships between ETL and RPE parameters appear to slightly strengthen when ETL are adjusted to individual fitness capacities, with special emphasis on cardiorespiratory fitness (MAS).
Ramos et al., 2019 (48) *Observational study*	Under 17 (*n* = 14), Under 20 (*n* = 14) and adult (*n* = 17) international women soccer players	High intensity (HID) = 15.6–20 km·h^−1^ Sprint > 20 km·h^−1^	Likely to almost certainly differences among all age brackets for the HID and sprint were found (adult > U20 > U17, ES varying from 0.41 [20.23–1.06] to 3.69 [2.63–4.76]), except for the comparison between U17 and U20 for sprint where the differences were rated as unclear. HID: adult (756 m) > U20 (688 m) > U17 (485 m). Sprint: adult (307 m) > U20 (223 m) ≈ U17 (192 m).
Asian-Clemente et al., 2020 (49) *Observational study*	Under 19 professional male soccer players from an elite Spanish first division soccer club (*n* = 17) SSGs (5c5c5 + 2) in 1 single 35 × 35 m pitch or in 2 28.5 × 28.5 m contiguous pitches (*n* = 4)	HSD = 18–21 km·h^−1^ VHSD > 21 km·h^−1^	VHSD (m·min^−1^): 2.5 ± 1.8 in 35 × 35 m, 12.8 ± 6.3 using 2 contiguous 28.5 × 28.5 m pitches, 4.6 ± 2.3 in official matches. When soccer is played in smaller relative areas than those used for official games, the ACC and DEC will be increased. Similarly, forcing players to change spaces quickly during SSGs promotes greater running activity, with higher HSD and VHSD covered per player. Although most of the running demands during matches were simulated with the proposed SSGs, it may be necessary to design other types of tasks to train for peak speed and distance covered at sprint speed.
Kelly et al., 2020 (50) *Observational study*	English Premier League male players (Manchester United) (*n* = 26) Entire season (*n* = 1)	HSD > 14.4 km·h^−1^ VHSD = 19.8–25.2 km·h^−1^	HSD was greater 3 days before a game (MD-3) vs MD-1 (95% CI, 140–336 m) while VHSD was greater on MD-3 and MD-2 than MD-1 (95% CI range, 8–62 m; *p* < 0.001). HSD was similar between mesocycles during the whole season suggesting that training schedules employed in elite soccer may be highly repetitive likely reflecting the nature of the competition demands.
Scott et al., 2020 (51) *Observational study*	Elite female players from National Women's Soccer League (NWSL, United States) (*n* = 36) Match observations (*n* = 208, 11 ± 6 per player)	HSR ≥ 12.5 km·h^−1^ or 60% vIFT (50% PS) VHSR ≥ 19 km·h^−1^ or 80% vIFT (65% PS) Sprint ≥ 22.5 km·h^−1^ or 30% ASR (80% PS)	Subjective ratings of fatigue and wellness are not sensitive to substantial within-player changes in match physical performance. HSR, VHSR, and SR thresholds customized for individual players athletic qualities did not improve the dose-response relationship between external load and wellness ratings. PS in elite women = 30.5 ± 1.8 km·h^−1^ (mean of 5 different roles). Match demand (ABS): HSR = 2,401 ± 454 m; VHSR = 398 ± 143 m; SR = 122 ± 69 m.
Altmann et al., 2021 (52) *Observational study*	German Bundesliga male players (*n = 25*) Match observations (*n* = 163)	HID = 17.0–23.99 km·h^−1^ Sprint ≥ 24.0 km·h^−1^	CM showed both the largest total (11.66 ± 0.92 km, ES = 0.68–1.86) and HID (1.57 ± 0.83 km, ES = 0.08–0.84) compared to all other positions, WM demonstrated the largest sprinting distance (0.42 ± 0.14 km, ES = 0.34–2.39). Some professional soccer players will likely incur differences in the composition of physical match performance when switching positions and therefore should pay special consideration for such differences in the training and recovery process of these players.
Oliva-Lozano et al., 2022 (53) *Observational study*	Spanish LaLiga male players (*n = 277*) Match observations (*n* = 1,252)	Maximal Intensity Sprint: when an acceleration occurred from 14 km·h^−1^ and the player got to exceed 30 km·h^−1^ for 0.2 s.	Professional soccer players need to be prepared for maximal intensity sprints in the first period of the match as well as maximal intensity sprints under high fatigue conditions given the frequency of sprints in the last period of the match. Training drills should be designed with a special focus on non-linear sprints without possession of the ball, based on the main tactical purpose of each position (e.g., CD: interceptions; CM: recovery runs; FB, WM and FW: run the channel).

ABS, absolute thresholds; ACC, accelerations; ACWR, acute:chronic workload ratio; AMP, average metabolic power; ASR, anaerobic speed reserve [MSS – MAS]; AU, arbitrary units; CB, central backs; CD, central defenders; CF, central forwards; CM, central midfielders; CoD, change of direction; DEC, decelerations; ES, effect size; ETL, external training load; FB, full-backs; FW, forwards; GK, goalkeepers; HID, high-intensity distance; HIR, high-intensity running; HMLD, high metabolic load distance; HR, heart rate; HRDP, heart rate deflection point; HSD, high-speed distance; HSR, high-speed running; LSG, large sided game; MAS, maximal aerobic speed; MD −/+ n, match day minus/plus n days, i.e., n days before/after the match; MIP, maximum intensity period; MSG, medium sided game; MSR, moderate speed running; PS, peak speed; RD, running distance; RPE, rating of perceived exertion; SR, sprint running; sRPE-TL, session rating of perceived exertion training load; SSG, small sided game; TD, total distance; TMr, training/match ratio; UEFA, Union of European Football Associations; VAM-EVAL, a modified version of the Montreal track test; VHSD, very high-speed distance; VHSR, very high-speed running; VHIR, very high-intensity running; vIFT, final velocity of the 30:15 intermittent fitness test; WM, wide midfielder.

Four studies were carried out with female players, 25 with male players and 1 with both female and male players. These studies were carried out between 2013 and 2022 and comprised a total of 1,897 participants, divided as follows: 97 adult females and 1,800 adult males. The total number of analyzed games was 442 for females and 2,098 for males. The asymmetry between the number of players and the number of games is due to the different objects of the studies. The male sample takes into account both training monitoring and matches, while the female sample includes only data collected during matches. The total number of pre-season and in-season weeks was 287 overall. The total number of single drills analyzed was 209. The key outcomes of the selected studies in this systematic review included velocity thresholds definition, match demands and training outcomes in terms of HSR and sprint distance.

## Discussion

The aims of this systematic review were: (1) to summarize the evidence on velocity thresholds used to classify HSR and sprinting in adult professional female and male soccer players, (2) to examine the existing evidence about the use of individualized thresholds, (3) to describe the HSR and sprinting demands during soccer matches, and (4) to provide training strategies for eliciting HSR and sprinting during training sessions in professional adult soccer. The main findings were: (1) non-standard and a large range of thresholds are used to monitor HSR and sprinting demands among professional soccer players; (2) absolute and relative thresholds could be used to analyze or compare performances across players and to monitor training at the individual near-to-maximum velocities, respectively; (3) HSR and sprint distances are position-dependent as well as highly variable across the phases of the game; (4) the combination of contextualized game-based and running-based drills should be used to ensure adequate HSR and sprinting exposure during training.

### Defining “absolute” thresholds: high, very high and sprint running distance

**Figure 2:**
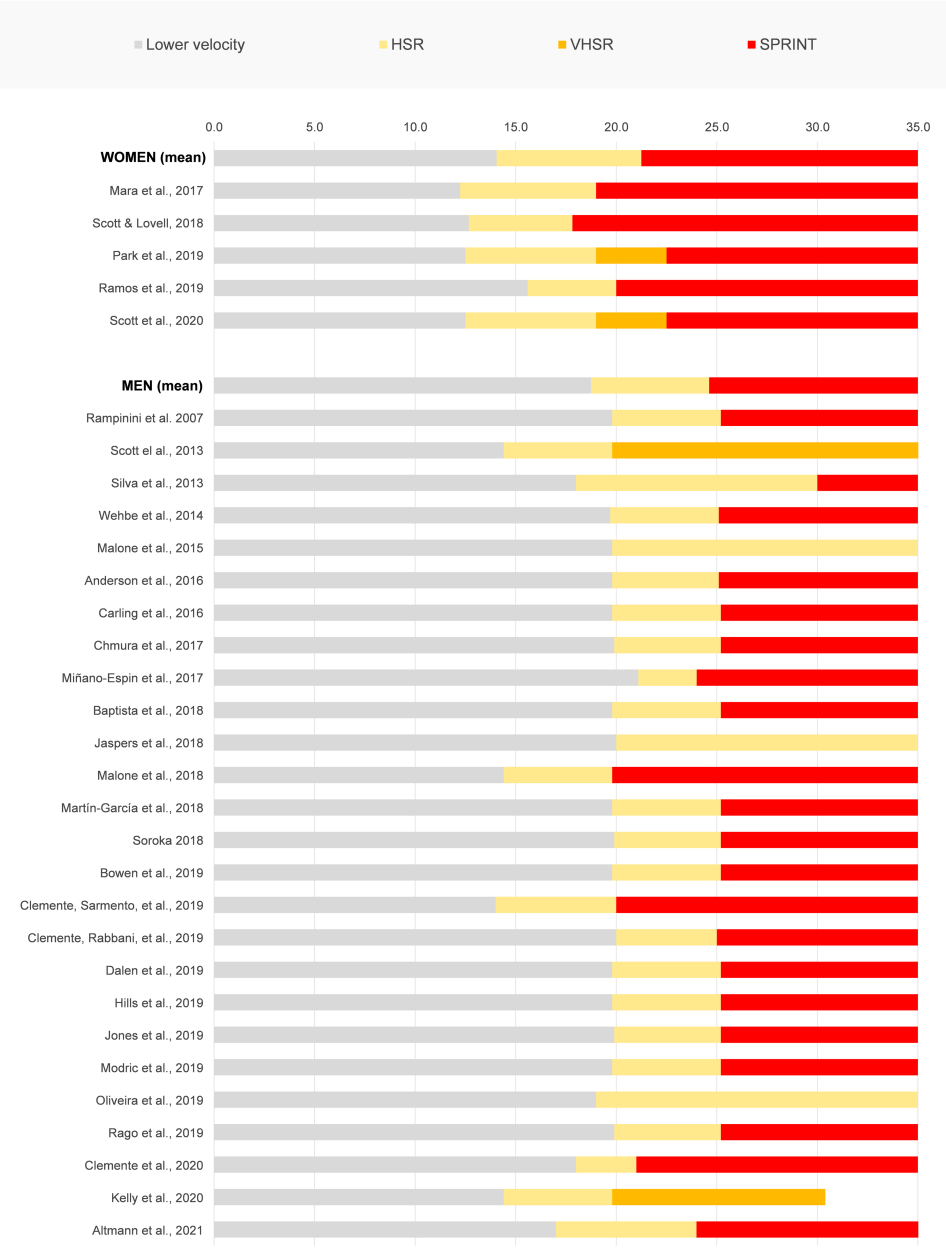
High-speed running (HSR), very high-speed running (VHSR) and sprint thresholds for elite adult female and male soccer players expressed in km·h^−1^.

To date, there is no consensus in the soccer literature about standard thresholds defining zones of running intensities (19). Figure 2 shows the range of velocity thresholds used in the studies conducted on professional adult female and male soccer players that were included in this systematic review. *High-speed running*, *high-intensity distance* and *high-speed distance* entry velocity are usually set between 12.2 km·h^−1^ and 15.6 km·h^−1^ for females, and between 14.4 km·h^−1^ and 21.1 km·h^−1^ for males, with the most common HSR entry velocity being 12.5 km·h^−1^ and 19.8 km·h^−1^ for female and male, respectively. Similarly, sprint distance entry velocity is commonly set between 17.8 km·h^−1^ and 22.5 km·h^−1^ (22.5 km·h^−1^ was the most common) for females and between 19.8 km·h^−1^ and 30 km·h^−1^ (25.2 km·h^−1^ was the most common) for males. This clearly shows the large variability in velocity for the same external load metrics commonly used among soccer scientists and practitioners.

Two studies used three different thresholds to define running in female soccer: *high-speed*, *very high-speed* (VHSR), and *sprint running* velocity (46, 51). Specifically, Park et al. developed an approach based on logical validity and analysis rigor by using a spectral clustering technique with application of a *β* = 0.1 smoothing factor to compute the exact velocity thresholds for the analysis of external load data collected from international female soccer players. The authors were able to define velocity thresholds as follows: HSR ≥ 12.5 km·h^−1^, VHSR ≥ 19 km·h^−1^, sprint ≥ 22.5 km·h^−1^ (46). Scott et al. reported the use of the same thresholds based upon the final outcomes in the 30:15 intermittent fitness test (vIFT) in terms of peak velocity reached by the players: HSR ≥ 12.5 km·h^−1^ or 60% vIFT (∼50% peak velocity), VHSR ≥ 19 km·h^−1^ or 80% vIFT (∼65% peak velocity), Sprint ≥ 22.5 km·h^−1^ or 30% anaerobic speed reserve (∼80% peak velocity) (51). The same results coming from these two studies seem to support the robustness of the proposed thresholds for adult female soccer, albeit further investigation is warranted.

Similar to the approach reported above, data mining modeling was proposed to define standard definitions and thresholds for male players by Dwyer and Gabbett 2012. The actual average distribution of velocities was calculated and series of Gaussian normal curves representing four velocity ranges was computed for best fit. The intersecting points for each Gaussian curve were used to determine the velocity range for each of the following locomotive activities: walking, jogging, running and sprinting with the entry velocity for sprinting determined at 21.35 km·h^−1^. While the conceptual operationalization and the robustness of this approach appear rigorous, the threshold definition emerging from this study could be questioned due to the very low sample analyzed (5 games of 5 players in a professional Australian A-League team), low sample frequency of the GPS units utilized (i.e., 1 Hz), and the lack of evidence suggesting that the velocities within each zone follow a Gaussian distribution (54). To our knowledge no other attempts to establish the rational for the use of “absolute” thresholds on male players were conducted using sufficiently rigorous methods. Therefore, based on the current literature, although these approaches sound promising, the definitions of the thresholds for HSR, VHSR and sprint are still arbitrary (55, 56) with no consensus in the soccer literature (see Figure 2).

In addition to the lack of agreement about absolute thresholds to be used, practitioners have to consider that the physical performance level of soccer players continuously improves. For these reasons, it seems desirable for sports scientists to have the capacity to adjust the velocity thresholds and to reprocess the collected data, especially when comparing or sharing data with clubs and federations adopting different numerical references. This approach seems a viable and practical solution at least until consensus on the definition of standard velocity thresholds is achieved. We believe that the establishment of an international standard (by practitioners and manufacturers) may facilitate the data exchange between clubs and national teams, which in turn could increase the value of velocity monitoring in soccer. We suggest that technology providers allow practitioners to set their absolute thresholds (this is indispensable for comparisons with historical data owned by the club) and to provide default international standardized thresholds, which could be used to share data with other clubs or national teams. Even if this is achieved, practitioners need to be aware that some limitations in accuracy and reliability exist between tracking technologies (e.g., between GNSS brands), therefore caution is needed when data from different clubs (that use different devices) are compared (57).

### Relative velocity thresholds

The use of individualized thresholds quantifying internal load measures [i.e., heart rate, maximum oxygen consumption (VO_2max_)] can facilitate training prescription and monitoring by setting relative work intensities corresponding to individual physiological targets (58). For example, coaches and sport scientists can tailor the training plans based on well-defined physiological parameters such as VO_2max_, maximum heart rate and onset of blood lactate accumulation (OBLA) (59). What has just been described above could also be used for the evaluation of individual external load parameters such as running velocity. The rationale of implementing relative thresholds for velocity parameters is justified by the assumption that absolute thresholds fail to account for the players' individual physical capacities, and therefore, they could result in an inappropriate assessment of the players' external load performed during training (21) and matches (55). Practitioners should consider that players have specific physical characteristics (e.g., peak velocity) that should be accounted for during the monitoring of training and matches. The use of relative individual thresholds would allow for more precise programming of the training load, which could help to design the appropriate dose of HSR and sprinting distance, preventing the implementation of unattainable velocities that could potentially be injurious (60), or not high enough to elicit the desired adaptation (61, 62).

Previous research has tried to individualize specific velocity thresholds based on physiological or performance parameters using some tests, which have been summarized in this review. HSR was defined as the velocity corresponding to the VO_2max_ [maximal aerobic speed (MAS)] in both women (63) and men (47), which was assessed through gas analysis methods during an incremental ramp test or the final velocity reached during the Yo-Yo Intermittent Recovery Test level 1 (64, 65). Alternatively, HSR threshold was set at the velocity corresponding to the heart rate deflection point determined from an incremental field test in women (35) or from a different incremental field test in men (60). When considering players' physical and fitness attributes, sprint entry velocity was defined as the velocity corresponding to the MAS determined from an incremental field test in female players (35), or as the value ≥30% of the anaerobic speed reserve, calculated as the difference between maximal sprint velocity and MAS in male players (47). For further details about maximal sprint speed and anaerobic speed reserve definition, the reader is invited to refer to the article of Buchheit and Laursen (66) and Sandford et al. (67).

Nevertheless, the validity criterions underpinning the determination of individual velocity thresholds using physiological parameters collected during continuous test protocols rather than external load proxies fail to consider the intermittent and repeated accelerative profile, which is typical of soccer (68), and as such seems inappropriate or at least inaccurate.

In contrast with the physiological approaches reported above, another common method to define relative thresholds from measures of external load is the percentage of the individual peak velocity, measured as the maximal velocity attainable during an all-out effort (69). Using this rationale, sprint running entry velocity was set at 80%–85% of peak velocity reached in a >30 m sprint test in female players (63) and at 80% of peak velocity reached in a 40 m sprint test in male players (60). In another study, sprint threshold was set either at >80%, >85% or >90% of the highest running velocity measured during either training sessions or matches (70). To date, the most reliable and simplest procedure to determine the peak velocity is through GNSS systems during a 40 meters sprint test (18, 63, 69). Alternatively, peak velocity can be tracked and determined from official matches (71), although this approach is not exempt from limitations due to the fact that players do not necessarily always reach maximal velocities during matches due to the contextual constraints and their specific positional demands (18, 69). In official matches some between-gender differences were observed for sprint velocities with 30.5 ± 1.8 km·h^−1^ (mean of 5 different roles) (51) and 32.0 ± 1.0 km·h^−1^ (mean of 3 different roles) (72) for female and male players, respectively. In consideration of the accuracy and reliability of tracking devices (14) now easily affordable and widely available, it would be reasonable to perform, at the beginning of a training session and after a standardized warm-up procedure, an all-out 30–40 m sprint test as a valid, high ecological and time-efficient approach to determine peak velocity for every player, whereby individual velocity thresholds could then be defined.

Although the number of studies that support the concurrent validity of the use of individualized thresholds are limited, we still have some evidence, specifically previous studies have reported the association between internal load and HSR demands. The perceptual responses (RPE using Borg's category ratio scale - CR10) provided by soccer players (Italian Serie B) at the end of the match were moderately correlated (*r* = 0.53–0.59) to distance covered expressed using absolute velocity thresholds ranging between 14.4 and 19.8 km·h^−1^ and HSR (>19.8 km·h^−1^) (47). Notably, the strength of the correlations tended to increase, albeit not significantly, when individualized velocity thresholds were used (*r* = 0.58–0.67) (47). Moreover, distance covered by sprinting was moderately correlated to RPE only when an individualized threshold was used (*r* = 0.55) (47). In contrast, the use of individualized velocity thresholds were not able to better quantify the dose-response of female soccer players during a 21-day training camp (35). This study reported that the quantification of the external load using players' peak sprinting velocity demonstrated a lower capacity to determine the dose-response of training, with consistently lower associations with heart rate and RPE (35). In another study, HSR and sprinting thresholds customized for individual female players athletic qualities did not improve the dose-response relationship between external load and wellness ratings (51). In summary, the individualization of velocity parameters based on players' individual fitness level (i.e., MAS or peak velocity) only marginally improves (trivial or small magnitude of the change) relationships between external and internal training load parameters (35, 47, 51). Based on the evidence reported so far, it is possible to confirm that internal and external training load parameters, independently by the use of absolute and relative thresholds, are different constructs and for this reason practitioners should monitor both.

The current evidence does not allow us to make definitive conclusions about the use of individualized velocity thresholds in soccer. While the use of individualized thresholds seems to offer the advantage of a more precise quantification of the individual external load, it may preclude comparisons between players, between training sessions and matches or within time when the same players have changed their individual velocity thresholds (73). In our opinion, either absolute or relative velocity thresholds seem appropriate to monitor HSR and sprinting exposure in professional soccer players. While absolute values are suitable to make between-player comparisons, relative thresholds are preferable for the individualization of the high-velocity aspects of the external training load. However, more research is needed on this topic before recommending the use of one over the other.

### High-speed running and sprinting during official matches

A summary of HSR and sprinting distance outcomes and related velocity thresholds during matches among professional adult female and male soccer players is reported in Table 3. HSR (>15.6 km·h^−1^) and sprint (>20 km·h^−1^) demands in professional female soccer were around 1,000 m (range: 911–1,063 m, 10.1–11.8 m·min^−1^) and 270 m (range: 223–307 m, 2.5–3.4 m·min^−1^), respectively. In professional male soccer players, the analogous outcomes for HSR (>19.8 km·h^−1^) and sprint (>25.1 km·h^−1^) demands were around 760 m (range: 618–1,001 m, 6.9–11.1 m·min^−1^) and 200 m (range: 153–295 m, 1.7–3.3 m·min^−1^).

**Table 3:** High-speed running (HSR) and sprint match demands for elite adult female and male soccer players.

Studies	Subjects	HSR		Sprint	
Mara et al. 2017	Women – Elite Australian	*12.2*–*19 km·h^−1^*	2,452 m	*>19 km·h^−1^*	615 m
Scott et al. 2020	Women – Elite United States	*≥12.5 km·h^−1^*	2,401 m	*≥22*.*5 km·h^−1^*	122 m
Ramos et al. 2019	Women – Adult	*15.6*–*20 km·h^−1^*	**756 m**	*>20 km·h^−1^*	**307 m**
Ramos et al. 2019	Women – U20	*15.6*–*20 km·h^−1^*	**688 m**	*>20 km·h^−1^*	**223 m**
Anderson et al. 2016	Men – Premier League	*19.8*–*25.1 km·h^−1^*	**706 m**	*>25*.*1 km·h^−1^*	**295 m**
Modric et al. 2019	Men – Elite Croatian	*19.8*–*25.1 km·h^−1^*	**462 m**	*>25*.*1 km·h^−1^*	**156 m**
Carling et al. 2016	Men – League 1	*19.8*–*25.2 km·h^−1^*	**587 m**	*>25*.*2 km·h^−1^*	**184 m**
Kelly et al. 2020	Men – Premier League	*19.8*–*25.2 km·h^−1^*	620 m	*–*	–
Miñano-Espin et al. 2017	Men – La Liga	*21.1*–*24.0 km·h^−1^*	277 m	*>24 km·h^−1^*	247 m
Wehbe et al. 2014	Men – Elite Australian	*>19*.*7 km·h^−1^*	**645 m**	*–*	–
Baptista et al. 2018	Men – Elite Norwegian	*≥19*.*8 km·h^−1^*	**744 m**	* *	
Rampinini et al. 2007	Men – League 1	*>19*.*8 km·h^−1^*	**821 m**	*–*	–
Stevens et al. 2017	Men – Eredivisie	*>19*.*8 km·h^−1^*	**738 **m	* *	
Dalen et al. 2019	Men – Elite Norwegian	*>19*.*8 km·h^−1^*	**747 m**	*>25*.*2 km·h^−1^*	**153 m**
Clemente et al. 2019	Men – Dutch and Spanish 2nd Division	*>20 km·h^−1^*	**730 m**		
Asian-Clemente et al. 2020	Men – U19 elite Spanish	*>21 km·h^−1^*	414 m	*–*	–
Altmann et al. 2021	Men – Bundesliga	*17.0*–*23.99 km·h^−1^*	1,340 m	*≥24 km·h^−1^*	495 m

Data are grouped by HSR zone to facilitate between-studies comparison. Bold values were considered for mean match demand calculation reported in the text.

Female soccer players perform a large proportion of high-speed runs (12.24–19.0 km·h^−1^) and sprints (>19.0 km·h^−1^) over distances shorter than 10 m (81%–84% and 71%–78%, respectively), with an average recovery time of 14 s between high-speed runs and 87 s between sprints, i.e., a 1:7 and 1:43 work to rest ratio, respectively (30). Similarly, in professional male players the most common distance covered in HSR (≥19.8 km·h^−1^) was 1–5 m, apart from the full backs who covered average HSR runs between 6 and 10 m (33).

Practitioners need to consider that the between-match variability for HSR (19.8–25.2 km·h^−1^) and sprint (>25.2 km·h^−1^) distances is notably high and is affected by the positional role (29, 52). Higher variability has been reported for central players (midfielders and defenders) while lower variability for wide midfielders and attackers (29, 74, 75). For example, the CV for female players ranged between 28% and 41% for HSR (>16.3 km·h^−1^) and between 35% and 65% for sprint (>20.0 km·h^−1^) distance (74). In male professional players, the CV for HSR and sprint ranged between 16% and 18% and between 31% and 37% respectively (29, 75). Moreover, the characteristics of HSR and sprints differed between positional roles and period of the match (30). In the 2010 World Cup, the largest amount of HSR (19.9–25.2 km·h^−1^) and sprint (>25.2 km·h^−1^) distance was observed in midfielders (38), which did not completely reflect the outcomes of previous studies conducted in the English Premier League and Spanish Primera Division in 2006–2007, where strikers were found to cover the largest sprint distances (76). In addition, practitioners should consider that the main tactical purpose of each playing position influence how the player has to perform maximal intensity sprints: interceptions for central defenders, recovery runs, closing down and pressing for midfielders, running in the channel to receive/exploit space, break into the box, or run-in-behind for wide-midfielders and forwards (53). Moreover, when conducting a contextual analysis of the physical demand during matches, HSR and sprinting seem to be affected by the quality of the opposition, with increasing values reported during matches played against stronger than weaker opponents (72). Moreover, a further level of contextualization requires interpreting these findings in consideration of the result of the game. In fact, independently from the opponents' level, it seems that soccer players perform significantly less high-intensity activity (21.1–24.0 km·h^−1^) when winning than when losing or when the score is balanced (31). This may be the main reason why no differences were found in the distances covered by players of Real Madrid (that won during the explored period approximately 70% of the total matches played) depending on the strength of the opposing team (31). Another common scenario in professional soccer and worthy of consideration pertains to fixture congestion. From preliminary results, it seems that playing many consecutive games does not affect the amount of HSR (19.9–25.2 km·h^−1^) covered during the consecutive matches (43), although the flawed methodological approach to quantify HSR exposure across studies investigating this area precludes to make definitive conclusions (77).

Considering the average match demands as the only reference could mislead strategies aiming at physically preparing players during training. In 2014 World Cup, the mean HSR (19.9–25.2 km·h^−1^) distance covered across all positions was 8.8% ± 2.1% of the total distance, but with midfielders peaking at roughly 17% (10). Interestingly, relying upon the most intense periods of the game, relevant consideration for training prescription can unfold. For example, the mean and peak HSR distances (>14.3 km·h^−1^; 24.6 and 47.4 m·min^−1^, respectively) doubled when considering 5-min epochs in Australian-league soccer (26). In Norwegian players, HSR (>19.8 km·h^−1^) and sprinting (>25.2 km·h^−1^) in the most demanding 5-min epochs reached 19 ± 3.5 and 8.8 ± 4 m·min^−1^ respectively, while the match mean reported in the same study was 8.3 ± 2.1 and 1.7 ± 0.7 m·min^−1^, respectively (41). In the Spanish La Liga, analyzing high-metabolic demands by using 1-min epochs revealed 49.9 ± 19.8 and 16.6 ± 17.4 m·min^−1^ for HSR (>19.8 km·h^−1^) and sprinting (>25.2 km·h^−1^), respectively (37). In view of these reference values, it sems reasonable to consider higher benchmark values to not underestimate the real exercise intensity during matches or when planning the prescription of training drills aiming at exposing soccer players to HSR and sprint distances. However, practitioners should consider that the “maximal intensity period” is a complex and composite construct reflecting an extreme internal response elicited *via* various combinations of physical and contextual factors. To note, this demands do not occur concurrently during the game and similarly for all metrics and players (78), thus a more accurate analysis of “maximal intensity period” requires a case-by-case approach.

### High-speed running and sprinting during training

High-speed running and sprinting distances are the metrics with the highest variability observed across days during the weekly training microcycle (between 60%–120%), and higher as compared to official matches (between 20%–30%) (36). This variability is reasonably a consequence of the weekly plan that requires a day-by-day load modulation and especially the unpredictable fluctuating nature of game-based drills such as sided-games. These findings may partially occur due to specific and different positional demands which are exacerbated during game-based training drills. Therefore, game-based drills should be implemented in combination with other forms of training to mitigate the large variability in terms of HSR and sprinting. Moreover, individual HSR and sprinting cumulative distances and frequency should be monitored to ensure effective load management strategies, especially to avoid detraining for those players less taxed during the game.

Knowledge of the match physical demands allows for the development of appropriate prescription of the training load as to adequately prepare individual players. Summaries from studies involving elite (40, 45, 79, 80) and sub-elite professional players (36, 81) revealed that for total distance and accelerations the training to match ratios tend to vary from ∼1 to 4 arbitrary units (AU) (that means in 1 week of training players were exposed to 1–4 times the match-load), with the exceptions of the ratios for HSR and sprinting distance, which were relatively lower and clearly under-attained during the training week compared to other measures such as total distance and accelerations (see Table 4). For HSR the training to match ratio was reported to vary between 0.2 AU and 2.3 AU, while for sprinting the values ranged from 0.03 AU (i.e., trivial sprinting exposure during training) to 1.3 AU. Remarkably, these ratios are average team values, which in consideration of the large inter-subject variability observed for the same external load metrics should be interpreted with caution.

**Table 4:** Training/Match ratio (T/M ratio) for high-speed running (HSR) and sprint in adult male soccer players.

Reference	Subjects	HSR weekly load				Sprint weekly load			
		Thresholds	Training	Match	T/M ratio	Thresholds	Training	Match	T/M ratio
Anderson et al. 2016	Men – Premier League	*19.8*–*25.1 km·h^−1^*	156	706	0.2	*>25*.*1 km·h^−1^*	8	295	0.03
Kelly et al. 2020	Men – Premier League	*19.8*–*25.2 km·h^−1^*	987	620	1.6	* *			
Clemente, Rabbani et al. 2019	Men – Elite Portuguese	*20*–*24.9 km·h^−1^*	–	–	2.3	* *			
Stevens et al. 2017	Men – Eredivisie	*>19*.*8 km·h^−1^*	811	738	1.1	* *			
Martin Garcia et al. 2018	Men – La Liga - Reserve	*>19*.*8 km·h^−1^*	726	440	1.7	*>25*.*2 km·h^−1^*	131	100	1.3
Baptista et al. 2018	Men – Elite Norwegian	*≥19*.*8 km·h^−1^*	460	744	0.6	*≥25*.*2 km·h^−1^*	69	144	0.5
Clemente, Owen et al. 2019	Men – Dutch and Spanish 2nd Division	*>20 km·h^−1^*	1,342	730	1.8				

Only data referred to weeks within 4 or 5 training days + 1 match day are reported. Data are grouped by HSR zone to facilitate between-studies comparison

In view of the current evidence, particular attention should be directed towards non-starting players as recent studies conducted in the Italian Serie A and the English Premier League revealed that non-starting players were exposed to considerable lower HSR and sprinting distances as compared with starting players (21, 82). Accordingly, it seems reasonable that dedicated compensatory drills targeting HSR and sprinting should be implemented during training to compensate for the lack of match related HSR and sprint running exposure and to avoid detraining. To design specific sprint training drills, playing position and contextual variables should be considered, for instance, defenders usually sprint to intercept the ball, midfielders run to close down and press the opponents, and attackers run throughout the channel to receive/exploit space and break into the box (53).

When soccer players use smaller relative areas during training compared with those used for official games, the number of accelerations and decelerations increase, but it is difficult to achieve adequate volumes of HSR (83). For instance, matches are played on a 105 × 68 m pitch (i.e., 357 m^2^ per player) that allow for an HSR distance of 8.4 m·min^−1^ and sprinting distance of 2.2 m·min^−1^, while during SSG 4v4 using a pitch of 39 × 39 m (i.e., 190 m^2^ per player), the HSR distance is of 2.7 ± 0.9 m·min^−1^ and sprinting distance of 0.1 ± 0.1 m·min^−1^, and during medium sided games (6v6) played on pitch of 47 × 43 m (i.e., 168 m^2^ per player) the HSR distance is 3.7 ± 2.1 m·min^−1^ and sprinting distance of 0.2 ± 0.5 m·min^−1^ (41). Instead, sided-games designed as large formats and with relative areas per player greater than 225 m^2^ and 300 m^2^ seem adequate to induce HSR and sprint distances, respectively, comparable to the analogous match external load outcomes (84). However, it is worth noting that the uncontrolled and unpredictable nature of game-based approaches may still cause large variability across players with the risk of overexposure to some and underexposing to others.

An alternative or complementary training method to sided-games to induce HSR and sprinting exposure are running-based drills with linear and non-linear sprints. Again, starting from the performance model defined by the game, strength and conditioning coaches should consider that the mean sprint (>30 km·h^−1^) duration recorded in LaLiga players ranged from 5 to 9 s, with a mean distance covered ranging from 30 to 55 m (53). Mixing linear sprints and sided-games, Ade and colleagues implemented repeated runs lasting 15 s and performed by young under 19 soccer players immediately before and after a sided-games bouts to ensure adequate coverage of distances above 19.8 km·h^−1^ (85, 86). In under 19 elite male players, asking players to change zone of the pitch quickly during small sided-games promoted higher HSR covered per minute. These authors compared a ball possession drill played in a single pitch (35 × 35 m pitch) to a drill with 2 contiguous pitches (28.5 × 28.5 m each), and they found that HSR was 2.5 ± 1.8 m·min^−1^ in the single pitch (i.e., 72 m^2^ per player) and 12.8 ± 6.3 m·min^−1^ using 2 contiguous pitches, while during official matches was 4.6 ± 2.3 m·min^−1^ (49). Another option to perform HSR and sprinting distance is to use isolated running-based drills or adding running phases during sided-games. In this case, HSR and sprint running exposure can be accurately prescribed and controlled with a lower degree of uncertainty given that the running intensity is predetermined, fixed, and easily monitored.

More recently, a game profile-based training (GPBT) approach has been proposed to induce relative HSR and sprint running distances comparable or greater than matches outcomes in under 19 elite male soccer players (87). A GPBT could be defined as 1 or more bouts of physical and technical activities (e.g., high-intensity intermittent running, changes of direction, and passes), which replicate the type of movements and physical demands (e.g., internal and external loads) of match-play (88). It was reported that a GPBT was more demanding in terms of distance run above 19 and 25.2 km·h^−1^ compared with a 5v5 small sided-game in a 42 × 30 m pitch (i.e., 126 m^2^ per player), specifically, 10.2 m·min^−1^ during GPBT vs. 4.6 m·min^−1^ during small sided-game for HSR and 4.2 vs. 2.0 m·min^−1^ for sprinting (87). Moreover, beneficial chronic effects on linear sprinting capabilities over 10 m and 20 m were found following a 8-week training period including GPBT, with greater improvements compared to sided-games training in the form of 5-a-side formats. While generalizing such findings to other cohorts warrants caution, the nature of the GPBT drills as fixed running circuits entailing intermittent phases of walking, jogging, running and sprinting may presume that similar outputs can be expected among adult female or male soccer players as well.

Another aspect to be considered when preparing players for HSR and sprint running game demand is sprinting in fatigue condition, since maximal intensity sprints were reported to be more frequent in the first, but also in the last, 15 min of the match, regardless of the playing position (53). Training HSR and sprint running at the end of the training session should therefore be taken into consideration even if a higher risk of musculoskeletal injury is conceivable.

In summary, practitioners are recommended to use a combination of adapted sided-games, GPBT, and running-based drills to ensure adequate HSR and sprint running exposure to their players during training. HSR and sprinting exposition are particularly important for non-starting players that need to compensate for missing the speed load exposition of the match, which often demands near-to-maximal velocity efforts (21, 82).

## Limitations and future directions

A number of limitations should be acknowledged in regard to this review: (1) inclusion of studies published in English only, which may have excluded relevant evidence on the topic coming from other languages; (2) some possible methodological issues (e.g., statistical power and confidence in the result) could arise because some studies have a relatively small sample size, while only 5 out of 30 studies included a large cohort; (3) only 5 out of 30 studies involved female professional players, mainly due to the lower diffusion of soccer among women; (4) this study did not analyze in depth the effect of technical and tactical factors on HSR and sprint demands during matches; (5) considering that the training information (related to HSR and sprint demands) reported in this review comes from studies that have mainly enrolled youth players instead of senior players (i.e., first-team players), this study cannot fully generalize the main findings to adult professional cohorts. As such, future studies should address these limitations and focus their attention on the monitoring of HSR and sprint demands during training among adult male and female (who are particularly underrepresented in the extant literature) professional players.

## Practical applications

Since there is no consensus on a specific absolute threshold defining high-speed running and sprint in adult female and male soccer players, and currently an international standard for such velocity thresholds does not exist, practitioners could set as entry velocity for HSR and sprinting values included in the range suggested from this review. A second option for practitioners is to use the velocity thresholds (HSR and sprint) adopted by FIFA and UEFA such as 19 km·h^−1^ and 23 km·h^−1^ for female and 20 km·h^−1^ and 25 km·h^−1^ for male.

Beyond absolute velocity thresholds, relative thresholds should be considered for specific training sessions where the goal is to reach near to maximal velocity exposure accounting for players' individual physical velocity capacity.

When analyzing the match demand, practitioners should consider that HSR and sprint distances are position-dependent as well as highly variable across the phases of the game and between the games: using HSR and sprint distance as performance indicators could introduce bias if not contextualized. In any case, players have to be ready for HSR and sprinting: to train the HSR and sprinting game demand, practitioners could use a combination of adapted sided-games, GPBT, and running-based drills to ensure adequate HSR and sprint running exposure to their players during training. Finally, monitoring HSR and sprint distances during every single session can allow the practitioner to verify the validity of the training process and optimize physical development, which is necessary to carry out the most demanding phases of the game, which require velocities close to the maximum (e.g., during high-speed counterattack).
