# Corrigendum: High-speed running and sprinting in professional adult soccer: current thresholds definition, match demands and training strategies. A systematic review

**DOI:** 10.3389/fspor.2023.1323440

**Published:** 2023-11-06

**Authors:** Antonio Gualtieri, Ermanno Rampinini, Antonio Dello Iacono, Marco Beato

**Affiliations:** ^1^ Sport Science and R&D Department, Juventus Football Club, Torino, Italy; ^2^ School of Health and Sports Sciences, University of Suffolk, Ipswich, United Kingdom; ^3^ Human Performance Laboratory, MAPEI Sport Research Centre, Olgiate Olona, Italy; ^4^ Sport and Exercise Discipline Group, Human Performance Research Centre, Faculty of Health, University of Technology Sydney, Moore Park, NSW, Australia; ^5^ Division of Sport and Exercise, School of Health and Life Sciences, University of the West of Scotland, Hamilton, United Kingdom

**Keywords:** football, GNSS, GPS, velocity thresholds, team sports, elite sports

A Corrigendum on High-speed running and sprinting in professional adult soccer: Current thresholds definition, match demands and training strategies. A systematic review By Gualtieri A, Rampinini E, Dello Iacono A and Beato M. (2023) Front. Sports Act. Living 5:1116293. doi: 10.3389/fspor.2023.1116293

**Additional Affiliation(s)**

In the published article, there was an error regarding the affiliation for Ermanno Rampinini. As well as having affiliation 3, he should also have:

Sport and Exercise Discipline Group, Human Performance Research Centre, Faculty of Health, University of Technology Sydney, Moore Park, New South Wales, Australia.

The authors apologize for this error and state that this does not change the scientific conclusions of the article in any way. The authors apologize for this error and state that this does not change the scientific conclusions of the article in any way. The original article has been updated.

